# Exogenous application of salicylic acid improves freezing stress tolerance in alfalfa

**DOI:** 10.3389/fpls.2023.1091077

**Published:** 2023-03-09

**Authors:** Xia Wang, Jiamin Miao, Wenjuan Kang, Shangli Shi

**Affiliations:** College of Grassland Science, Gansu Agricultural University, Lanzhou, China

**Keywords:** candidate genes, freezing stress, *Medicago sative*, salicylic acid, signal transduction

## Abstract

Freezing stress is one of the most detrimental environmental factors that can seriously impact the growth, development, and distribution of alfalfa (*Medicago sativa* L.). Exogenous salicylic acid (SA) has been revealed as a cost-effective method of improving defense against freezing stress due to its predominant role in biotic and abiotic stress resistance. However, how the molecular mechanisms of SA improve freezing stress resistance in alfalfa is still unclear. Therefore, in this study, we used leaf samples of alfalfa seedlings pretreatment with 200 μM and 0 μM SA, which were exposed to freezing stress (-10°C) for 0, 0.5, 1, and 2h and allowed to recover at normal temperature in a growth chamber for 2 days, after which we detect the changes in the phenotypical, physiological, hormone content, and performed a transcriptome analysis to explain SA influence alfalfa in freezing stress. The results demonstrated that exogenous SA could improve the accumulation of free SA in alfalfa leaves primarily through the phenylalanine ammonia-lyase pathway. Moreover, the results of transcriptome analysis revealed that the mitogen-activated protein kinase (MAPK) signaling pathway-plant play a critical role in SA alleviating freezing stress. In addition, the weighted gene co-expression network analysis (WGCNA) found that *MPK3*, *MPK9*, *WRKY22* (downstream target gene of *MPK3*), and TGACG-binding factor 1 (*TGA1)* are candidate hub genes involved in freezing stress defense, all of which are involved in the SA signaling pathway. Therefore, we conclude that SA could possibly induce *MPK3* to regulate *WRKY22* to participate in freezing stress to induced gene expression related to SA signaling pathway (NPR1-dependent pathway and NPR1-independent pathway), including the genes of non-expresser of pathogenesis-related gene 1 (*NPR1)*, *TGA1*, pathogenesis-related 1 *(PR1)*, superoxide dismutase (*SOD)*, peroxidase *(POD)*, ascorbate peroxidase (*APX)*, glutathione-S-transferase *(GST)*, and heat shock protein *(HSP)*. This enhanced the production of antioxidant enzymes such as SOD, POD, and APX, which increases the freezing stress tolerance of alfalfa plants.

## 1 Introduction

Alfalfa (*Medicago sative* L.) is one of the most important forage crops cultivated worldwide (Chen et al., 2021c), whereas the extreme environmental conditions can seriously impact alfalfa growth and development, especially sudden freezing stress, not only reducing biomass productivity but also restricting the geographical distribution of alfalfa (Chen et al., 2015; Adhikari et al., 2021; Song et al., 2021). Therefore, it is important to study the mechanisms regulating freezing stress responses in alfalfa.

Freezing stress can cause negative changes at physiological, biochemical, and molecular levels, and preliminarily leads to inhibition of photosynthetic activity, ion permeability, nutrition uptake (Huang and Cheung, 2021), increased oxidant stress due to Reactive oxygen species (ROS) accumulation and induced redox homeostasis and other changes (Cui et al., 2019). At low temperatures, the formation of ice crystals in plants directly result in cellular dehydration and membrane damage (Knight and Knight, 2012). Plants have evolved numerous regulatory mechanisms to cope with the damage caused by freezing stress (Shu et al., 2017). To scavenge ROS, plants trigger their antioxidant system to synthesize antioxidant enzymes, including superoxide dismutase (SOD), peroxidase (POD), ascorbate peroxidase (APX), catalase (CAT), and glutathione reductase (GR) (Wang et al., 2009a; Dong et al., 2014; Zhuo et al., 2018; Zaid and Wani, 2019). To survive, plants have evolved precise thermos sensory system to trigger signal transductions and provoke numerous freezing-related genes to improve freezing tolerance (Ding et al., 2022). Signal transductions include the calcium signal transduction and the mitogen-activated protein kinase (MAPK) signal module. Örvar et al. (2000) found that low temperatures stimulated calcium signal transduction in alfalfa. MAPK cascades have been reported to play an important role in regulating downstream biotic and abiotic stress-related genes (Chen et al., 2021a), including MAP kinase kinase kinase (MAPKKK), MAP kinase kinase (MPKK), and MAPK.

In addition, freezing stress can increase endogenous phytohormones, including abscisic acid (ABA), polyamines (PAs), and SA, which enhances freezing tolerance (Zhang et al., 2011). SA is a natural phenolic phytohormone and is a vital hormone that participates in numerous plant physiological processes, including seed germination, vegetative growth, nodulation in legumes, and stomatal closure. Furthermore, SA plays a critical role in the resistance to biotic and abiotic stress, such as freezing stress (Ding et al., 2016; Khokon et al., 2017; Wassie et al., 2020). In plants, there are two distinct pathways to generate SA: one is the isochorismate synthase (ICS) pathway, which is in the chloroplast. Isochorismate is synthesized from chorismic acid and transforms to SA *via* isochorismate pyruvate layse (Dong et al., 2014). Previous reports have found that the *ICS1/ICS2*, Enhanced disease susceptibility *(EDS)5*, and GH3.12/avrPphB susceptible 3 (*PBS3*) genes play a critical role in the ICS pathway of SA biosynthesis, and that 90% of SA was synthesized through the ICS pathway during pathogen attacks (Ding and Ding, 2020; Mishra and Baek, 2021). The second pathway is phenylalanine ammonia-lyase (PAL). In the cytoplasm, the phenylalanine is deaminated by PAL and converted to trans-cinnamic acid, while the other bifurcate is oxidized to trans-cinnamic acid and converted to benzoic acid (BA). BA is hydroxylated by BA 2-hydroxylase (BA2H) to synthesize SA (Dong et al., 2014). Interestingly, *PAL* genes in *Arabidopsis* play a vital role in the PAL pathway to against environmental stress (Huang et al., 2010). Although multiple pieces of evidence demonstrated that those two pathways were activated under abiotic stress, it is unclear how they have been activated and regulated by the related genes. With increasing researches have assessed the mechanism of SA defense against biotic and abiotic stress, the SA mechanism in plant pathogen resistance is well known. In *Arabidopsis*, SA binds to receptor non-expresser of pathogenesis-related gene 1 (NPR1) to regulate pathogenesis-related (PR) genes and other SA-induced genes to resist disease (Wang et al., 2020a; Chen et al., 2021b). Since NPR1 has no DNA binding domain, Zhou et al. (2000) demonstrated that NPR1 can activate PR-1 genes through TGACG-binding motif (TGA) 2 and TGA3 binding to the activating sequence-1 (as-1) element. Fan and Dong (2002) demonstrated that overexpressed TGA2.2 decreased SA- and PR-related genes in tobacco, while TGA2 is an SA-responsive and NPR1-dependent activator in tobacco. Thus, TGA interacts with NPR1 differently in different species.

Some WRKY genes binding to the W-box motif (TTGACC/T) participated in regulating the NPR1-dependent PR genes to resist pathogen attacks, except for TGAs (Chen et al., 2021b). The *WRKY18*, *WRKY40*, and *WRKY60* triple mutant increased the expression of PR-1 genes to resist bacterial pathogen attacks (Xu et al., 2006), *WRKY53*, *WRKY54*, and *WRKY70* were expressed in active downstream-related genes against pathogen infections (Wang et al., 2006; Zhou et al., 2018). WRKY could be phosphorylated and inactivated by a MAPK cascade during a pathogen attack, which would depend on MAPK and control the activity and subcellular location of *WRKY22* and *WRKY29* (Asai et al., 2002). Activating the expression of *AtMPK4* could reduce the accumulation of SA to against *Pseudomonas syringae* pv tomato strain DC3000 (Pst DC3000) (Liang et al., 2013), while the MEKK1-MKK1/2-MPK4 cascade negatively regulated genes based on MEKK1 or MPK4, which decreased SA and PR genes (Rodriguez et al., 2010). Automatically, MAPK cascades are not only mediated by freezing stress but also by SA signaling. In addition to the NPR1-dependent pathway, SA also induced the expression of glutathione peroxidase (*GPX*, Li et al., 2013), glutathione-S-transferase (*GST*, Blanco et al., 2005), heat shock proteins (HSPs, Jumali et al., 2011), *POD*, *SOD*, and *APX* (Per et al., 2017) as NPR1-independent pathway-related genes to enhance antioxidant and non-antioxidant activities against stresses. Intriguingly, Olate et al. (2018) reported that the signaling of cold and pathogen response was crosstalk, including the SA signal pathway. Automatically, PRs as important genes in SA signal pathway also play an important role in freezing stress. However, it remains unknown how SA application improves freezing stress resistance (Yeh et al., 2000; Griffith and Yaish, 2004). Recently, numerous researches have reported that exogenous SA could improve the freezing tolerance through physiological changes in many plants (Saleem et al., 2021b), including alfalfa (Miura and Tada, 2014). And Wang et al. (2022) have found that SA could play a vital role in the freezing stress of alfalfa leaves, while the mechanism of SA in freezing stress requires further investigation.

In our study, we tested the effect of SA on the freezing resistance of alfalfa and performed a transcriptome analysis to explore the molecular mechanism of SA under freezing stress. Our data reveal that pretreatment of SA could protect alfalfa from freezing stress and proposed a working model on how SA might regulate freezing stress to tolerance in alfalfa. This study contributes to a better understanding of the molecular mechanisms of SA-induced alfalfa freezing tolerance, which provides a cost-effective way of reducing freezing stress damage in alfalfa.

## 2 Material and methods

### 2.1 Plant materials and treatments

Seeds of the alfalfa cultivar ‘WL326GZ’ (fall dormancy score 3.8) were obtained from Zhengdao Ecological Technology Co. (Beijing, China). The healthy seeds were sown on arenaceous quartz in a growth room at 20°C with 60%-80% humidity under a 14h light cycle with 200 μmol m^-2^ s^-1^ photosynthetic photons flux density and 10h dark at 18°C regimens. The seedlings were grown hydroponically in half-strength Hoagland solution, which was applied daily after the seeds germinated.

In this study, 200μM SA was used based on preliminary experiments that showed the most effective concentration to improve freezing stress. For SA application and abrupt freezing stress, three-week-old alfalfa seedlings were divided into two groups with WCK (control) and T (200μM SA treatment). The T group was watered with 20ml of SA solution with Ph 6-8 for five days before freezing stress while the WCK group was watered with 20ml pure water. Subsequently, the seedlings were transferred to a growth chamber at -10°C for 0h, 0.5h, 1h, and 2h for freezing stress. Finally, they were allowed to recover in a growth chamber with normal temperature (20°C/day, 18°C/night) for 2 days. A total of 30 leaf samples were harvested for use in this study. Detailed sample names are as follows: WCK01-03, WCK11-13, WCK21-23, WCK31-33, and WCK41-43 represent three samples of ‘WL326GZ’ exposed to -10°C at 0, 0.5, 1, 2 h, and recover for 2 days, respectively; T01-03, T11-13, T21-23, T31-33, and T41-43 represent three samples of SA treated ‘WL326GZ’ exposed to -10°C at 0, 0.5, 1, 2h, and recover for 2 days, respectively.

### 2.2 Enzyme extraction and assays

To investigate the effects of SA on antioxidant activities, we measured SOD, POD, and APX using the method described by Giannopolitis and Ries (1977) and Chance and Maehly (1955). First, we used fresh leaves of approximately 0.1g that were homogenized into a 2 ml tube with potassium phosphate buffer (50 mM, pH 7.0), and obtained the upper supernatant to measure the antioxidant activities *via* centrifugation for 20 min at 12000 r min^-1^ and 4°C. We then used the upper supernatant to measure the antioxidant activities using the method outlined by Wassie et al. (2020) with minor modifications.

### 2.3 Hormone assay content

To analyze changes in hormones in fresh leaves, we used high-performance liquid chromatography-mass spectrometry (HPLC-MS/MS) to measure the SA contents as described by Pan et al. (2010). Briefly, only 50 mg of each fresh sample was ground into a powder with liquid nitrogen and SA was twice extracted with acetonitrile to purify on the Poroshell 120 SB-C18 column (2.1×150,2.7 um). Finally, we measured the contents of SA by HPLC-MS/MS (Agilent 1290, SCIEX-6500Qtrap).

### 2.4 RNA extraction and Illumina sequencing

Total RNA was isolated from the leaf samples with a Trizol reagent kit (Invitrogen, Carlsbad, CA, USA) according to the manufacturer’s instructions. And thirty libraries were contrasted and sequenced using Illumina Novaseq6000 by Gene Denovo Biotechnology Co. (Guangzhou, China). To obtain high-quality clean reads, we used raw reads to remove reads containing adapters, more than 10% of unknown nucleotides, and more than 50% of low-quality (Q-value ≤ 20) bases through fastp (version 0.18.0) (Chen et al., 2018). Furthermore, we removed rRNA mapped reads, leaving only final clean reads for further assembly and calculation using the short reads alignment tool Bowtie 2 (version 2.2.8) (Langmead and Salzberg, 2012). Finally, paired-end clean reads were mapped to the alfalfa reference genome (https://figshare.com/projects/whole_genome_sequencing_and_assembly_of_Medicago_sativa/66380) using HISAT2. 2.4 with “-rna-strandness RF” and other parameters set as a default for further assembly (Kim et al., 2015).

### 2.5 Differential expression genes identification

For the quantity of each transcript’s expression and variations, we used FPKM (fragment per kilobase of transcript per million mapped reads) values and the RSEM software for calculations. Furthermore, the differential expression analysis was performed by DESeq2 between two different groups (WCK01-03 vs. WCK11-13, WCK01-03 vs. WCK21-23, WCK01-03 vs. WCK31-33, WCK01-03 vs. WCK41-43, T01-03 vs. T11-13, T01-03 vs. T21-23, T01-03 vs. T31-33, T01-03 vs. T41-43, WCK01-13 vs. T01-03, WCK11-13 vs. T11-13. WCK21-23 vs. T21-23 and WCK41-43 vs. T41-43). The transcripts with false discovery rate (FDR) < 0.05 and |log2 (fold change) | ≥1 were used as DEGs.

### 2.6 Gene function annotation

To analyze DEG functions, we used all DEGs mapped to Gene Ontology (GO) terms in the Gene Ontology database (http://www.geneontology.org/) to classify the genes to the three ontologies and significantly enriched (FDR ≤ 0.05) GO terms in DEGs, which were compared to the genome background and defined by a hypergeometric test. Furthermore, as the Kyoto Encyclopedia of Genes and Genomes (KEGG) is the major public pathway-related database outlining the genes’ biological function, we overlapped the DEGs with a pathway enrichment analysis and pathways with FDR ≤ 0.05 were defined as significantly enriched reactions in DEGs.

### 2.7 Weighted gene co-expression network analysis analysis, gene network co-expression, and visualization

WGCNA is a method of clustering modules of highly correlated genes among multiple genes. Co-expression networks were constructed using the WGCNA (v1.47) package in R, and 31,310 genes were used to analyze unsigned by WGCNA. However, the other parameter of WGCNA as power is 12, merge cut height is 0.75 and the minor module size is 50.

### 2.8 qRT-PCR analysis for candidate gene validation

To verify the efficacy of RNA-seq, we verified 6 genes by Quantitative Real-time PCR (qRT-PCR): MS.gene21548, MS.gene02035, MS.gene004340, MS.gene035685, MS.gene05280, and MS.gene017127. The cDNA was obtained from reverse transcription with the iScript cDNA Synthesis Kit (Bio-Rad Laboratories Inc., CA, USA) and the primers were designed by Primer 5.0, which is listed in **Table S1**. The qRT-PCR reaction was performed through Universal SYBR Green Master supermix (Roche, Shanghai, China), and then performed on the Applied Biosystems 7500/7500 fast Real-time PCR (BIORAD). There were three technical replicates of each sample. Finally, we used the 2^−ΔΔCt^ method to analyze the expression of all genes based using the UBL-2a as the reference gene (Castonguay et al., 2015).

### 2.9 Statistical analysis

The all data in our study were assessed by one-way analysis of variance (ANOVA) with SPSS 20.0 and performed in Excel 2019 software. The values represent the mean ± STEDV (n=3). Different letters indicate represent values that were significantly different between treatments at p<0.05. The networks of key modules and hub genes were visualized using Cytoscape v.3.3.0.

## 3 Results

### 3.1 SA treatment improved alfalfa survival under freezing stress and enhanced related antioxidant enzymes

**Figure 1:**
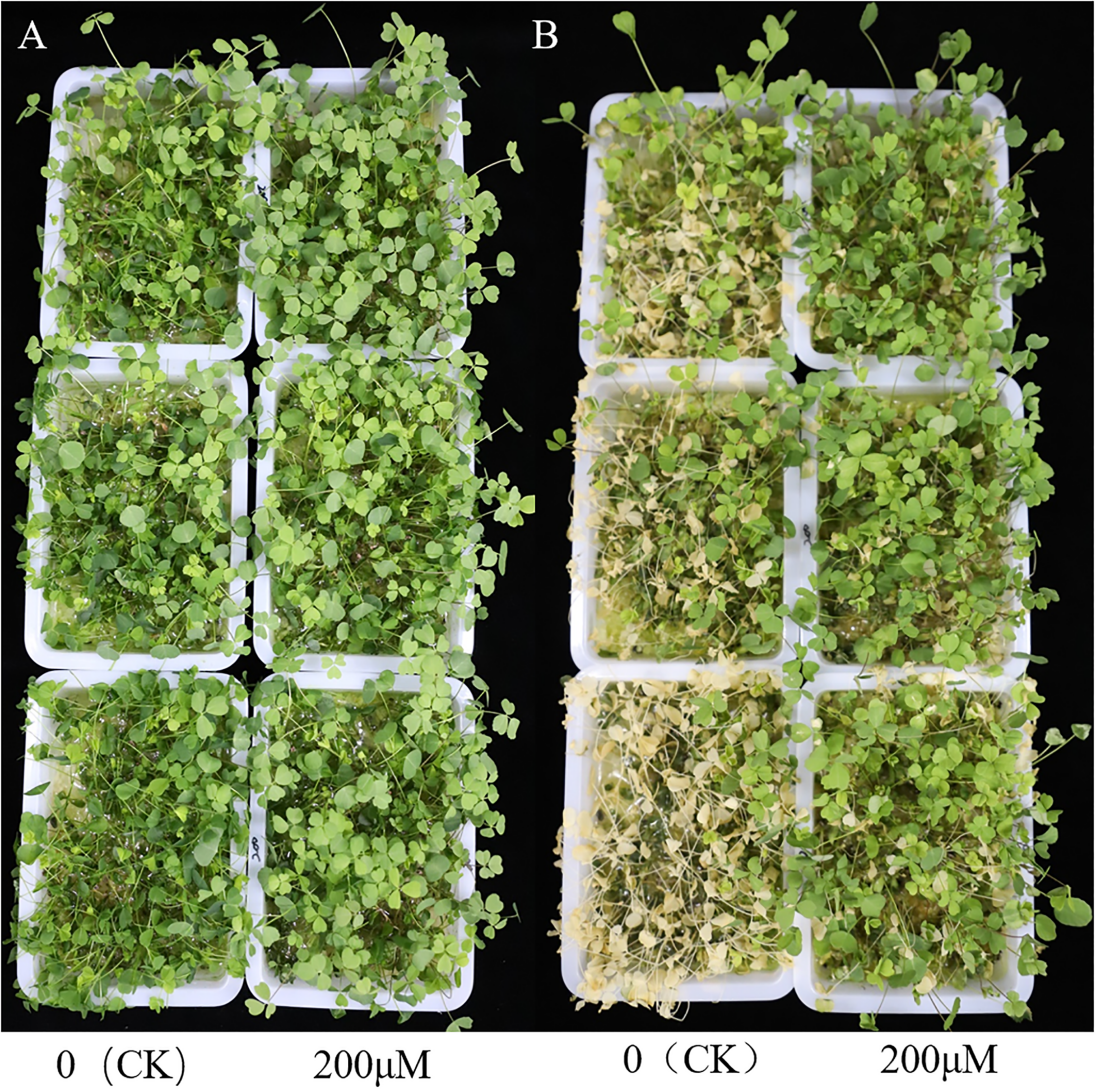
Phenotypes of 0 **(CK)** and 200 μM SA treatment alfalfa leaves to freezing stress. **(A)** The phenotype of 0 (CK) and 200 μM SA treatment “WL326GZ” leaves to -10°C for 2h. **(B)** The phenotype of the **(A)** growth under normal temperatures for 2 days.

To assess whether SA treatment improves alfalfa’s survival rate under freezing stress, we used 200 μM SA (treatment) and 0 μM SA (control) pretreatment on three-week-old alfalfa for five days, including exposure to -10°C for 0, 0.5, 1, 2h to observe changes in phenotypes. Obviously, alfalfa samples with 200μM SA treatment had a higher survival rate than the control under exposure to -10°C for 2h (**Figure 1A**). To exhibit the phenotypic changes, we subjected all alfalfa to growth chamber with normal temperature for 2 days (**Figure 1B**). Interestingly, 14.83% of treatment samples survived, while only 7.17% survived in the control group (**Figure 1B**), which indicates that SA treatment could improve alfalfa survival rate under freezing stress conditions.

**Figure 2:**
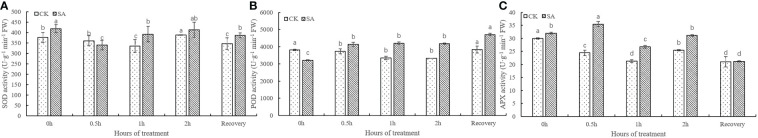
Effects of SA application on the activities of SOD **(A)**, POD **(B)**, and APX **(C)** in alfalfa leaves under freezing stress. The column chart represents the mean ± STEDV (n=3). The different letters represent values that were significantly different under CK or SA application to “WL326GZ” under freezing stress at different times (p<0.05, Duncan’s test).

To measure the effects of SA treatment on alfalfa leaves under freezing stress, we analyzed the antioxidant enzymes of SOD, POD, and APX (**Figure 2**). The results demonstrated that the changes in SOD activity between the CK and SA treatments dramatically decreased, and then significantly increased as the duration of freezing stress increased. Interestingly, the SOD activity of SA treatment was greater than in CK at different times except for exposure to freezing stress at 0.5h (**Figure 2A**). In the CK, POD activity dramatically decreased at 0.5h and there were no significant changes as the time of freezing stress increased. Similar to SA treatment, POD activity increased at 0.5h, and no remarkable changes were observed at different freezing stress times (**Figure 2B**). The variation in APX activity was similar to POD, but that of CK decreased dramatically at 0.5h and 1h (**Figure 2C**). All leaves of POD and APX activity of SA treatment were higher than in CK (**Figures 2B, C**). Therefore, SA treatment could enhance the leaves of SOD, POD, and APX activity enzymes to defend against freezing stress.

### 3.2 Identification of DEGs in different treatments and related DEGs, including GO functional annotation and KEGG pathway analysis

To explore the mechanism of SA treatment in alfalfa under freezing stress, we used RNA-seq to analyze all the leaf samples in this study. We generated 5,929,423,457-7,218,419,876bp clean data reads, which were cleaned by GC<42%, Q20>97%, and Q30>93% (**Table S2**). All raw data have been updated to National Center for Biotechnology Information (NCBI) under accession number PRJNA867517.

Furthermore, we identified 126,974 genes (**Table S3**) from clean reads after mapping to an alfalfa reference genome more than 93% (**Table S4**). Finally, we constructed thirteen comparisons to identify 41,175 DEGs, as seen in **Figure S1**. In detail, most DEGs were upregulated in WCK-0-vs-WCK-4 (7,957 DEGs) and T-0-vs-T-4 (7,601 DEGs), and most DEGs were downregulated in WCK-0-vs-WCK-3 (7,023 DEGs) and T-0-vs-T-3 (6,725 DEGs) (**Figure S1**).

To better understand the function of the DEGs, we mapped those DEGs to the GO database and the KEGG database (**Figure S2**). In WCK-0-vs-WCK-4, T-0-vs-T-4, WCK-0-vs-WCK-3, and T-0-vs-T-3, the results showed that the most abundant biological processes were metabolic processes and cellular processes; in cellular components, the most DEGs were enriched in the cell and cell part terms; in molecular function, the most DEGs were enriched in catalytic activity and binging terms (**Figure S2A1**–**Figure S2D1**). Meanwhile, in KEGG analysis, the DEGs are most enriched in the biosynthesis of the secondary metabolite pathway and metabolic pathways. There were some differences in DEGs enriched in the significant pathways. In WCK-0-vs-WCK-4, and T-0-vs-T-4, DEGs were significantly enriched in the biosynthesis of the secondary metabolite pathway and metabolic pathways. Similar to WCK-0-vs-WCK-3 and T-0-vs-T-3, DEGs were significantly enriched in the MAPK signaling pathway-plant, during biosynthesis of the secondary metabolite pathway and the plant-pathogen interaction pathway (**Figures S2A2**–**S2D2**). Therefore, it is possible that the MAPK signaling pathway-plants play a vital role in SA defense against freezing stress after alfalfa seedlings are exposed to -10°C for 2h.

### 3.3 Comparative analysis of DEGs in SA treatment at different times

**Figure 3:**
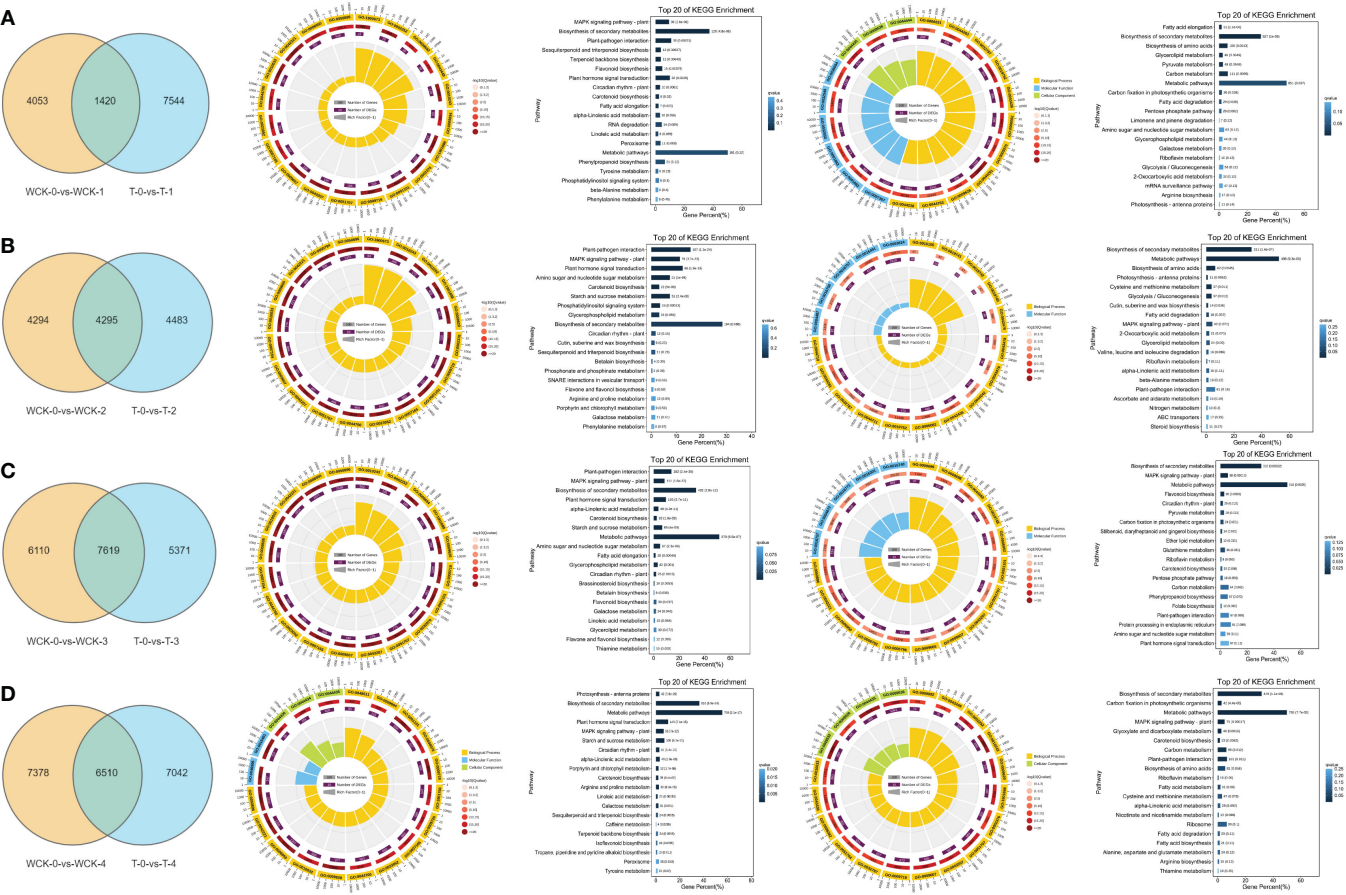
Comparison of DEGs between SA pretreatment and the control in alfalfa exposed to freezing stress for different periods. **A1, B1, C1**, and **D1** represent the Venn diagram of DEGs between SA pretreatment and the control in alfalfa exposed to freezing stress at 0.5, 1, and 2h, and allowed to recover for 2 days, respectively. **A2, B2, C2**, and **D2**; **A3, B3, C3**, and **D3** represent the common DEGs between SA pretreatment and control in alfalfa exposed to freezing stress at 0.5, 1, 2h, and allowed to recover for 2 days mapped to GO and KEGG database, respectively. **A4, B4, C4**, and **D4; A5, B5, C5**, and **D5** represent the especially DEGs of SA pretreatment alfalfa exposed to freezing stress at 0.5, 1, 2h, and allowed to recover for 2 days mapped to GO and KEGG database, respectively.

To explore the potential difference between SA treatment on DEGs and the control, we used 4 Venn diagrams to overlap the DEGs. The results showed that there were 1,420 DEGs in common between (WCK-0-vs Wck-1) and (T-0-vs-T-1) (**Figure 3A1**). In GO analysis, the circle diagram showed that the most DEGs of the top 20 most significant GO terms enriched were GO:0050896 (528, response to stimulus), GO:0042221 (382, response to chemical), and GO:0006950 (357, response to stress) (**Figure 3A2**). In KEGG pathway analysis, most DEGs were significantly enriched in the MAPK signaling pathway, and during biosynthesis of secondary metabolites and plant-pathogen interaction pathway (**Figure 3A3**). Moreover, there were 7,544 unique DEGs in (WCK-0-vs Wck-1) VS (T-0-vs-T-1), which could be due to SA treatment on the leaves. Most DEGs in the top 20 significant GO terms enriched were GO:0044238 (2845 primary metabolic process), GO:0044763 (2541, single-organism cellular process), and GO:0044444 (1852, cytoplasmic part) (**Figure 3A4**). The most significant KEGG pathway was enriched in the fatty acid elongation pathway, biosynthesis of secondary metabolites, and biosynthesis of amino acids pathway (**Figure 3A5**).

As in the freezing stress for 1h (WCK-0-vs Wck-2 VS T-0-vs-T-2), there were 4,295 DEGs in common between the SA treatment and control groups (**Figure 3B1**). In GO analysis, the most DEGs in the top 20 GO terms enrichment were enriched in GO:0050896 (1,431, response to stimulus), GO:0042221 (992, response to chemical), and GO:0050794 (906, regulation of cellular process) (**Figure 3B2**). In the KEGG pathway analysis, the most significant DEGs were enriched in the plant-pathogen interaction pathway, the MAPK signaling pathway-plant pathway, and the plant hormone signal transduction pathway (**Figure 3B3**). In addition to the shared DEGs, based on the SA application, there were 4,483 unique DEGs in this phase (**Figure 3B1**). The most DEGs of the top 20 significant GO terms were enriched in the GO:0003824 (1,819, catalytic activity), GO:0044710 (1,223, single-organism metabolic process), and GO:0044281 (652, small molecule metabolic process) (**Figure 3B4**). In the KEGG pathway, the most significant KEGG enrichment pathway was the biosynthesis of secondary metabolites, metabolic pathway, and biosynthesis of amino acids pathways (**Figure 3B5**).

As the duration of freezing stress increased, more DEGs (7,619) were shared between the SA treatment and control (WCK-0-vs WCK-3 VS T-0-vs-T-3 **Figure 3C1**). The most DEGs of the top 20 significant GO terms were enriched in GO:0050896 (2,438, response to stimulus), GO:0042221(1,614, response to chemical), and GO:0006950 (1,577, response to stress) (**Figure 3C2**). The 7,619 DEGs enriched in the KEGG of the top 3 pathways were plant-pathogen interaction, MAPK signaling pathway-plant, and biosynthesis of secondary metabolites pathways (**Figure 3C3**). Furthermore, there were 5,371 unique DEGs in the SA treatment group under freezing stress for 2h (**Figure 3C1**). Most DEGs of the top 20 significant GO terms coincided with shared DEGs, including GO:0050896 (1,460, response to stimulus), GO:0042221(888, response to chemical), and GO:0006950 (901, response to stress) (**Figure 3C4**). The results of the KEGG pathway analysis differed in metabolic pathways instead of the plant-pathogen interaction pathway (**Figure 3C5**).

Finally, we contrasted the DEGs in the WCK-0-vs Wck-4 VS T-0-vs-T-4. The results revealed there were 6,510 DEGs in common (**Figure 3D1**). The GO analysis results were kept within the unique DEGs in WCK-0-vs WCK-3 VS T-0-vs-T-3 (**Figure 3D2**). However, in the KEGG pathway analysis, the top 3 significant pathways were photosynthesis-antenna proteins, biosynthesis of secondary metabolites, and metabolic pathways (**Figure 3D3**). Based on the SA application, there were 7,024 unique DEGs in this comparison (**Figure 3D1**). The most DEGs of the top 20 significant GO terms were GO:0009536 (1,053, plastid), GO:0010033 (883, response to organic substance), and GO:0006082 (801, organic acid metabolic process) (**Figure 3D4**). In KEGG pathway analysis, the most significant DEGs were enriched in the biosynthesis of secondary metabolites, carbon fixation in photosynthetic organisms, and metabolic pathways (**Figure 3D5**).

Shared DEGs were highly related to freezing stress, and the terms GO:0050896 (response to stimulus), GO:0042221 (response to chemical), and GO:0006950 (response to stress) could play vital roles in freezing stress response, while the plant-pathogen interaction pathway, the MAPK signaling pathway-plant pathway, the plant hormone signal transduction pathway, and the biosynthesis of secondary metabolites pathways also play important roles in freezing stress. While the unique DEGs could have resulted in SA application, many GO terms interact to play a role in resisting freezing stress. In addition, the KEGG pathway of fatty acid elongation, biosynthesis of secondary metabolites, biosynthesis of amino acids, metabolic pathway, and MAPK signaling pathway-plant pathway could play critical roles in reducing freezing stress damage after SA application. Interestingly, the MAPK signaling pathway-plant pathway could play an important role in freezing stress and SA treatment to alleviate damage due to freezing stress.

### 3.4 Co-expression network analysis, GO and KEGG classification

**Figure 4:**
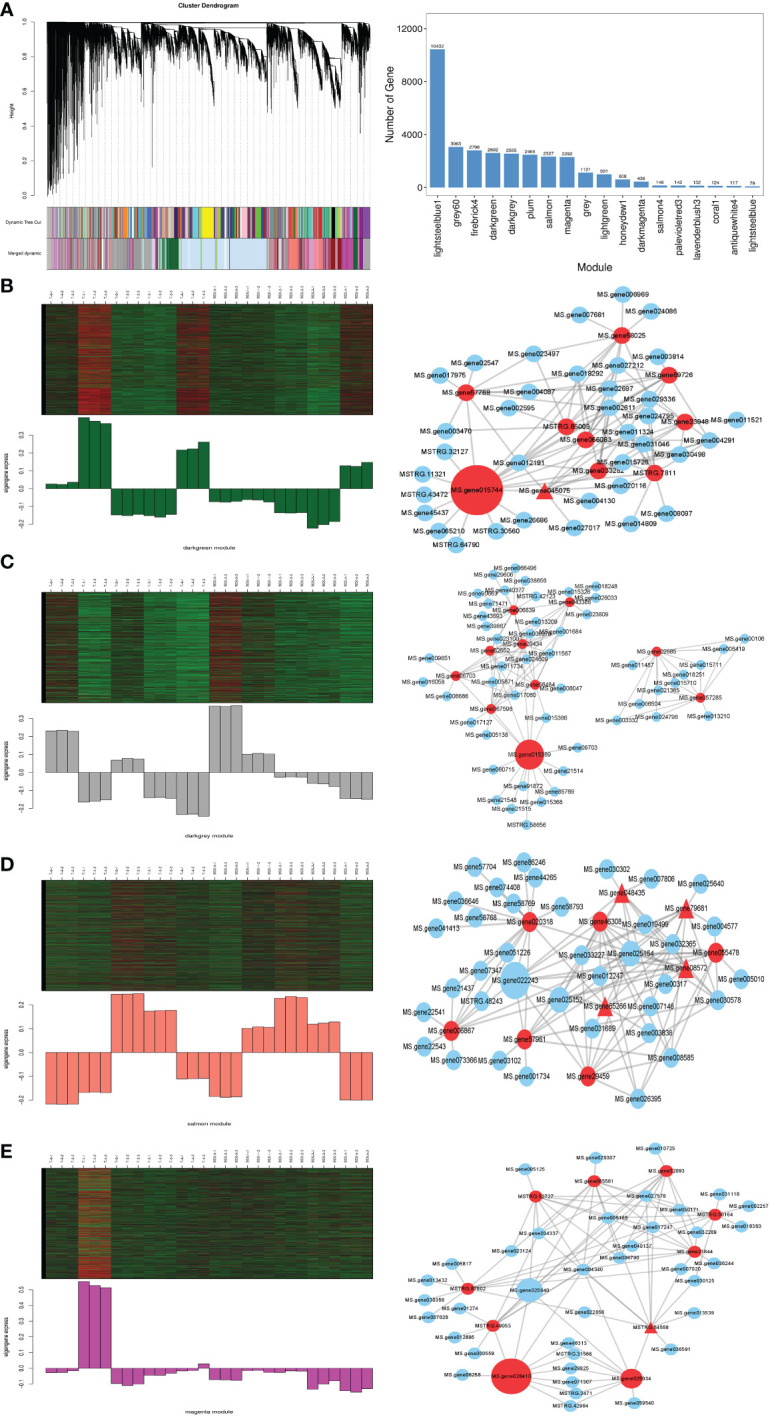
Weighted gene co-expression network analysis (WGCNA) of 31,310 genes. **(A1)** Cluster dendrogram; **(A2)** Number of genes of 18 modules; **(B1, C1, D1, E1)** The four modules of most related SA pretreatment alfalfa seedlings increased freezing stress; **(B2, C2, D2, E2)** The correlation networks of the top 10 hub DEGs corresponding to the four modules. The networks only showed the top 10 weight values of each hub gene, the different types of nodes represent the value of betweenness centrality is higher, the type of the node is bigger, and the triangle of the node represents the transcription factor.

To validate potential candidate genes related to SA application and reduce damage due to freezing stress, we chose 31,310 DEGs for WGCNA and divided them into 18 modules (Fig 4A1). Then we further analyzed the typical differences in expression modules at the same time as freezing stress. The results showed that the ‘darkgreen’ (2602), ‘darkgrey’ (2555), ‘salmon’ (2327), and ‘magenta’ (2292) modules were highly related to SA application under freezing stress (**Figure 4**). In the GO analysis (**Figure S3A1**), the most DEGs in the darkgreen module were enriched in the GO:0044464 cell, the GO:0005622 intracellular, and GO:0044424 intracellular parts. In KEGG pathway analysis (**Figure S3A2**), the most significant DEGs were enriched in the autophagy - other eukaryotes, ubiquitin-mediated proteolysis, and photosynthesis pathways.

In the darkgrey module, the most DGEs in GO terms were enriched in the GO:0044238 primary metabolic process, GO:0050896 response to stimulus, and GO:0051179 localization (**Figure S3B1**). Furthermore, in KEGG analysis (**Figure S3B2**), the DEGs were significantly enriched in the circadian rhythm – plant, ribosome biogenesis in eukaryotes, and plant hormone signal transduction pathways.

In the GO analysis in the salmon module (**Figure S3C1**), the results showed that the most DEGs were enriched in the GO terms of GO:0050896 (response to stimulus), GO:0042221 (response to chemical), GO:0006950 (response to stress). Moreover, in the KEGG pathway analysis (**Figure S3C2**), the DEGs were significantly enriched in the MAPK signaling pathway - plant, proteasome, and plant-pathogen interaction pathways.

In the magenta module, the GO analysis results showed that the most DEGs were enriched in the GO terms of GO:0044424 intracellular part, GO:0005737 cytoplasm, and GO:0044444 cytoplasmic part (**Figure S3D1**). In the KEGG pathway analysis, the DEGs were significantly enriched in the photosynthesis, ribosome, and photosynthesis - antenna proteins pathways (**Figure S3D2**).

To validate the candidate hub genes in those related modules, we used the All.kWithin value to measure the candidate genes in the module. The higher the value was, the more important the gene was in the module (**Table 1**). The candidate 10 genes of the four modules are shown in **Figure 4**, including MPK 3, MPK 9, and some transcription factors (transcription factor TGA1, transcription factor MYC2, WRKY transcription factor 22).

**Table 1:** The details of hub genes of four modules.

Gene ID	Module	All.kWithin	Symbol	Description
MSTRG.7811	darkgreen	211.23	CNOT9	CCR4-NOT transcription complex subunit 9 isoform X2 [Medicago truncatula]
MS.gene58025	darkgreen	184.89	SPS	sucrose-phosphate synthase [Medicago sativa]
MS.gene69726	darkgreen	180.04	VPS29	vacuolar protein sorting-associated protein 29 [Medicago truncatula]
MS.gene015744	darkgreen	176.66	ALDH2C4	aldehyde dehydrogenase family 2 member C4-like [Abrus precatorius]
MS.gene045075	darkgreen	175.60	TGA1	transcription factor TGA1 isoform X1 [Medicago truncatula]
MS.gene57768	darkgreen	166.73	CNGC5	probable cyclic nucleotide-gated ion channel 5 [Medicago truncatula]
MS.gene066083	darkgreen	161.86	TPS5	alpha,alpha-trehalose-phosphate synthase [UDP-forming] 5 [Medicago truncatula]
MS.gene23948	darkgreen	154.52	PLD1	phospholipase D alpha 1 [Medicago truncatula]
MSTRG.85009	darkgreen	152.18	At3g26720	alpha-mannosidase At3g26720 [Medicago truncatula]
MS.gene033282	darkgreen	148.15	RH30	DEAD-box ATP-dependent RNA helicase 20 [Medicago truncatula]
MS.gene56484	darkgrey	133.77	STP-1	alpha-1,4 glucan phosphorylase L-2 isozyme, chloroplastic/amyloplastic isoform X1 [Medicago truncatula]
MS.gene067596	darkgrey	127.17	STP-1	alpha-1,4 glucan phosphorylase L-2 isozyme, chloroplastic/amyloplastic isoform X1 [Medicago truncatula]
MS.gene20434	darkgrey	126.98	SS2	granule-bound starch synthase 2, chloroplastic/amyloplastic [Medicago truncatula]
MS.gene52552	darkgrey	126.66	MIOX2	inositol oxygenase 2 [Medicago truncatula]
MS.gene006839	darkgrey	119.90	STP-1	alpha-1,4 glucan phosphorylase L-2 isozyme, chloroplastic/amyloplastic isoform X1 [Medicago truncatula]
MS.gene06703	darkgrey	118.44	SS1	starch synthase 1, chloroplastic/amyloplastic isoform X1 [Medicago truncatula]
MS.gene043386	darkgrey	117.64	Mpv17l2	protein SYM1 [Medicago truncatula]
MS.gene015369	darkgrey	116.60	TIFY10A	protein TIFY 10a isoform X1 [Medicago truncatula]
MS.gene02685	darkgrey	114.89	FDH	beta-ketoacyl CoA synthase 10 [Medicago sativa]
MS.gene057285	darkgrey	114.77	KCS2	3-ketoacyl-CoA synthase 11 [Medicago truncatula]
MSTRG.54558	magenta	145.15	MPK9	mitogen-activated protein kinase 9 isoform X1 [Medicago truncatula]
MSTRG.87802	magenta	142.34	LSM2	sm-like protein LSM2 [Medicago truncatula]
MSTRG.49053	magenta	141.40	ALATS	alanine–tRNA ligase [Medicago truncatula]
MS.gene025934	magenta	131.93	UBC28	Ubiquitin-conjugating enzyme E2 [Zostera marina]
MS.gene21844	magenta	125.89	OASA1	cysteine synthase [Medicago truncatula]
MS.gene065561	magenta	120.15	PSBR	photosystem II 10 kDa polypeptide, chloroplastic [Medicago truncatula]
MSTRG.52727	magenta	119.69	ALATS	alanine–tRNA ligase [Medicago truncatula]
MS.gene028410	magenta	117.81	RPL44	60S ribosomal protein L44 [Medicago truncatula]
MS.gene32893	magenta	114.60	TAF4B	transcription initiation factor TFIID subunit 4b [Medicago truncatula]
MSTRG.56164	magenta	114.30	PAHX	phytanoyl-CoA dioxygenase domain protein [Medicago truncatula]
MS.gene57961	salmon	395.72	GAE1	UDP-glucuronate 4-epimerase 1 [Medicago truncatula]
MS.gene46308	salmon	392.05	AUX22E	auxin-induced protein 22e-like [Trifolium pratense]
MS.gene020318	salmon	385.57	AUX22E	auxin-induced protein 22e-like [Trifolium pratense]
MS.gene055478	salmon	380.16	GAE1	UDP-glucuronate 4-epimerase 1 [Medicago truncatula]
MS.gene65266	salmon	377.60	MPK3	LOW QUALITY PROTEIN: mitogen-activated protein kinase 3 [Medicago truncatula]
MS.gene006867	salmon	375.77	At4g26390	pyruvate kinase, cytosolic isozyme [Medicago truncatula]
MS.gene08572	salmon	375.66	MPK3	LOW QUALITY PROTEIN: mitogen-activated protein kinase 3 [Medicago truncatula]
MS.gene048435	salmon	366.46	MYC2	transcription factor MYC2 [Medicago truncatula]
MS.gene29459	salmon	356.49	CML23	calcium-binding protein CML24 [Medicago truncatula]
MS.gene79681	salmon	355.37	WRKY22	WRKY transcription factor 22 [Medicago truncatula]

kWithin Value: The gene connectivity within each module.

### 3.5 Analysis of candidate genes related to SA biosynthesis and content changes of SA in alfalfa

**Figure 5:**
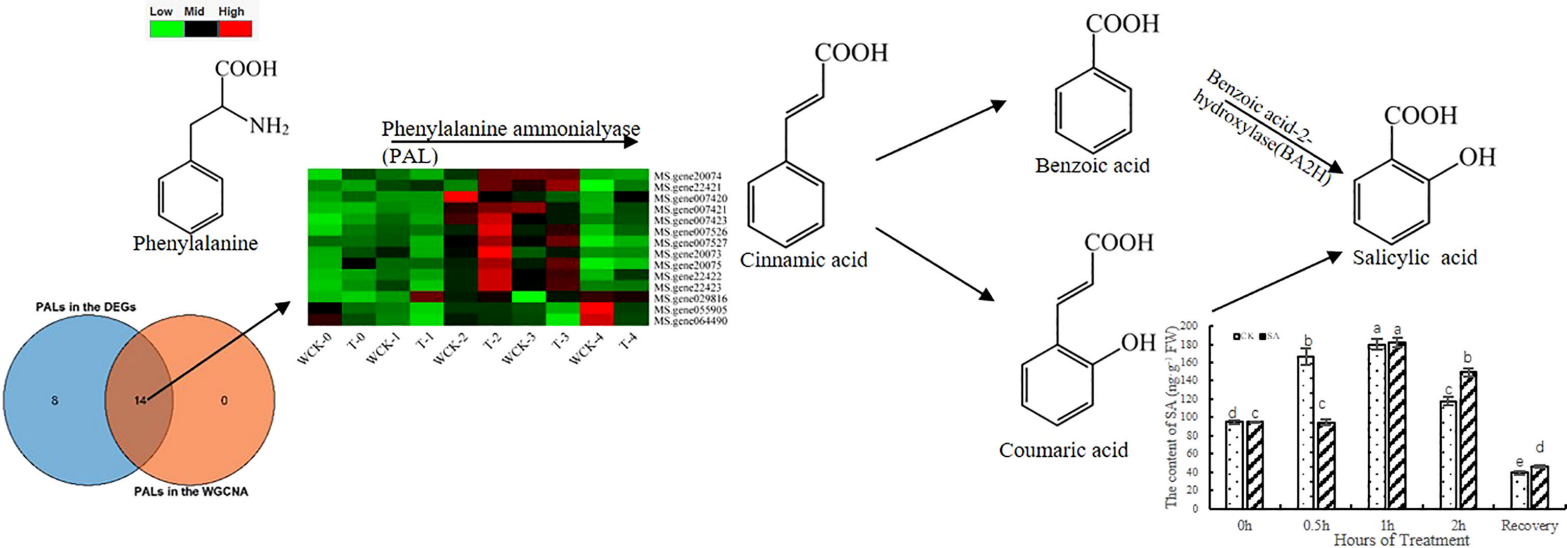
Analysis of the *PALs* expression related to increasing biosynthesis of SA.

In plants, there are two pathways related to the synthesis of SA *via* chorismite, which are a product of the shikimate pathway (Kumar, 2014). One pathway used cinnamate through PAL, and the other pathway used isochorismate (IC) through ICS. To analyze which pathway is dominant during freezing stress, we screened 14 *PALs*, 3 *ICSs* in the WGCNA analysis, and 22 *PALs* and 0 *ICSs* in all DEGs. Furthermore, we overlapped the genes to find out the 14 *PALs* from the WGCNA analysis were DEGs. The heatmap of the 14 *PALs* is shown in **Figure 5**. However, there were no *ICS* DEGs in our study, which suggests that the PAL pathway could predominate in the SA synthesis of leaves under freezing stress.

To analyze the changes in endogenous SA content, we used HLPC-MS/MS to detect the contents of all sample leaves. The results (**Figure 5**) showed that the SA content increased as the duration of freezing stress increased until 1h and then decreased. Interestingly, the content of CK increased earlier than in the exogenous SA. The CK could be activated earlier as a protection system to defend against freezing stress.

### 3.6 SA induced large numbers of genes in the SA signaling pathway to defend against freezing stress

In our study, there were 43 *MAPKKKs* (**Figure S4A**), 9 *MKKs* (**Figure S4B**), and 23 *MAPKs* (**Figure S4C**) that could play a role in transmitting SA signaling transduction. All genes reported in the NPR1-dependent pathway could participate in freezing stress. In the NPR1-dependent pathway, the application of SA during freezing stress induced the expression of 134 *WRKYs* (**Figure S4H**), and promoted the expression of 4 *NPR1* genes (**Figure S4D**). Interestingly, the expression of the *NPR1s* increased as the duration of freezing stress increased, and then decreased. There were 21 *TGAs* (**Figure S4E**), which as key NPR1 activate transcript factors, were also found in this experiment. Most *TGAs* in the SA application were more highly expressed than in the CK during freezing stress. Moreover, as the time of freezing stress increased, the expression of 11 *TRx hs* increased, and most *TRx h* genes in SA were more highly expressed than in the CK at 0.5 and 1h, while the opposite occurred at 2h (**Figure S4F**). The expression trend of 4 *PR1* genes was similar to *TRx h* genes, but the inflection point was at 0.5h of freezing stress. The expression of most *PR1* genes was higher than CK under freezing stress at 2h (**Figure S4G**).

Except for the NPR1-dependent pathway genes take a vital role against freezing stress, there have many NPR1-independent pathway genes expressed in this experiment, including *SOD*, *POD*, *APX*, glutathione S-transferases (*GST*), and *HSPs*. Fortunately, most of the 9 *SOD* genes were increased with the duration of freezing stress and then decreased, and the expression of *SOD* genes in SA application was higher than that of CK after freezing stress at 0, 0.5h, whereas opposite after freezing stress at 1 and 2h (**Figure S4I**). Among the 10 *POD* genes, except for the expression of *POD* genes in SA application after freezing stress at 2h were higher than that of CK, the others were lower than that of CK (**Figure S4J**). As in 12 *APX* genes, nearly most of them in SA application had higher expression than in CK after freezing stress at 0.5h, while the others were not more than in CK (**Figure S4K**). Moreover, SA and freezing stress could induce GSTs, and SA application had higher expression than CK during all freezing stress processes (**Figure S4L**).

High SA concentrations have been reported to induce HSPs (Jumali et al., 2011). In our research, there were 124 different types of HSP genes induced (**Figure S4M**), including *HSP 1* (4 genes), *HSP 15* (4 genes), *HSP 17* (8 genes), *HSP 18* (17 genes), *HSP 21* (2 genes), *HSP 22* (5 genes), *HSP 23* (1 genes), *HSP 70* (72 genes), *HSP 83* (5), *HSP 90* (6 genes). SA could induce HSP-related genes in freezing stress.

### 3.7 qRT-PCR validation of the expression profiles of the candidate genes

To validate the reliability of the transcriptome data, we randomly monitored the expression of 6 DEGs through qRT-PCR analysis: MS.gene 21548 (Rop-interacting receptor-like cytoplasmic kinase 2), MS.gene 02035 (regulatory protein NPR1 isoform X1), MS.gene 004340 (GDSL-like lipase/acylhydrolase), MS.gene 035685 (pathogenesis-related protein PR-1), MS.gene 05280 (mitogen-activated protein kinase 9), and MS.gene 017127 (mitogen-activated protein kinase 10 isoform X1) (**Figure S5**). Fortunately, the trends of gene expression by the qRT-PCR analysis were similar to the FPKM in transcriptome data analysis.

## 4 Discussion

### 4.1 SA effectively induced physiological response in leaves under freezing stress

SA is a potent phenolic signaling biomolecule and a highly potent plant growth regulator that can decrease crop biomass losses due to its cheap, biodegradable, and positive response to biotic and abiotic stresses (Arif et al., 2020; Zaid et al., 2022). Freezing stress is one of the most significant abiotic stresses that seriously impacts crop biomass (Barnaby et al., 2020), and can cause oxidative damage by excess ROS accumulation in plants (Wei et al., 2021). Pretreatment of SA in plants could increase the ability of the antioxidant system to defend against freezing stress (Arif et al., 2020). Similar to rice, soaking in SA solution increased the SOD, CAT, APX, and GR activities against freezing stress (Pouramir-Dashtmian et al., 2014), while Hossain et al. (2015) reported that the SA-induced antioxidant system decreases freezing stress by maintaining the integrity of the plasma membranes and other physiological functions. Simultaneously, exogenous 0.1 mM SA increased the activities of SOD, POD, and CAT under freezing stress in barley (*Hordeum vulgare* L.) (Mutlu et al., 2016). Interestingly, in our study, pretreatment of 200 µM SA increased SOD, POD, and APX activities against freezing stress (**Figure 2**), which was similar to rice and barley. Furthermore, Wang et al. (2012) reported that exogenous SA could improve the activities of CAT and POD at low temperatures, while the activity of SOD does not significantly change to CK. This could be because the SA pretreatment method is different. Therefore, exogenous application of 200 µM SA could improve the activities of SOD, POD, and APX in alfalfa under freezing stress.

### 4.2 SA induced differentially expressed genes in freezing stress

To elucidate the possible mechanism behind how SA application improved freezing stress in alfalfa, we transcribed RNA from alfalfa leaves pretreated with 0 and 200 µM SA, which were exposed to -10°C for 0, 0.5, 1, 2h, and allowed to recover to growth temperature for 2 days. We identified 126,974 genes by RNA-seq from the alfalfa reference genome (**Table S3**); these results were more effective than other research on alfalfa freezing stress (Song et al., 2016; Shu et al., 2017; Xu et al., 2019; Wang et al., 2021). To better understand the function of SA application in freezing stress, we constructed 13 comparisons to identify 41,175 DEGs for further analysis. The results demonstrated that the plant-pathogen interaction pathway, the MAPK signaling pathway-plant pathway, the plant hormone signal transduction pathway, and the biosynthesis of the secondary metabolites pathway could play a vital role in freezing stress (**Figure S2**). Wang et al. and Song et al. found that all pathways, except for the MAPK signaling pathway-plant that is predominant in alfalfa, confer freezing stress (Song et al., 2016; Wang et al., 2021). The MAPK signaling pathway has been reported as important in freezing signal transduction in many plants (Cowan and Storey, 2003; Liu and Zhou, 2018; Wu et al., 2022), which means it could have a similar function in our study. As for SA application, the domain KEGG pathways were fatty acid elongation, biosynthesis of secondary metabolites, biosynthesis of amino acids, metabolic pathway, and MAPK signaling pathway-plant. In bananas, Chen et al. (2020) found that SA could improve chilling stress *via* metabolic pathways and enhance energy charge by synthesizing fatty acids and amino acids (Chen et al., 2020). Many reports suggest that SA is involved in the induction of secondary metabolites (Khan et al., 2015), and thus pathway of biosynthesis of secondary metabolites pathway could be one of the domain pathways. SA is known as the primary signaling hormone that is always in coordination with other signal transduction - MAPK signaling pathways to participate in complex transduction networks to enhance abiotic tolerance in plants (Singh and Jwa, 2013; Chai et al., 2014; Liu et al., 2022). Moreover, the MAPK signaling pathway is dominant in defense signaling (Shan et al., 2021), and is involved in freezing stress and SA application.

### 4.3 MAPK cascades could play a dominant role in transmitting SA signals or SA-dependent related genes to defend against freezing stress

MAPK cascades are highly conserved signaling components in all eukaryotic organisms (Zheng et al., 2018) and play an indispensable role in plant growth, development, and biotic and abiotic stresses responses, (Mohanta et al., 2015), including in regulating hormonal responses (Long et al., 2020). MAPK cascades include MAPKKK, MPKK, and MPK (MAPK). In *Arabidopsis thaliana*, there were 60-80 MAPKKKs, 10 MAPKKs, and 20 MAPKs (Pitzschke, 2015; Saucedo-García et al., 2021). In our study, we identified 43 MAPKKKs, 9 MKKs, and 23 MAPKs as DEGs that belong to MAPK cascades (**Figure S4**). Moreover, the evidence showed that SA could induce BaMKK9, BaMPK1, 2, BaMKK2, 4, 5, and BaMPK3, 6 integrated into the SA signaling pathway to regulate defense genes in canola (*Brassica napus* L.) (Liang et al., 2013). AtMKK4/MKK5 is essential for the induction of SA-mediated defense response (Liu et al., 2021), while MPK3/MPK6 positively regulates SA-dependent defense responses (Bartels et al., 2009). Therefore, the MAPK cascades could take part in transmitting SA signaling or direct the SA-dependent related genes downstream against freezing stress.

We obtained three hub genes of SA application to alleviate the damage of freezing stress as MAPKs through WGCNA (**Table 1**). Two of them belong to MPK3, and the other is MPK9. Much research has found that MPK3 in different species plays an important role in SA mediating plant defense responses (Long et al., 2020). While AtMPK3 has been reported to direct regulated SA-dependent related genes against freezing defense and is related to SA biosynthesis (Bartels et al., 2009), Guan et al. (2020) revealed that NtMPK3 could interact with the SA signaling pathway in triclosan stress. Recently, evidence has found that MPK3 could mediate SA-dependent genes, such as PR-1 expression to defense response, and SA-independent genes to defense response (Genot et al., 2017). Consequently, the detailed function of the MPK3 remains unknown, though MPK3 occupied a predominant role in SA application against freezing stress. In addition, studies have demonstrated that MPK9 can be expressed in guard cells (Jammes et al., 2009) and positively regulated ABA signaling mediates stomatal closure (Liu et al., 2010). Moreover, Khokon et al. (2017) found that AtMPK9 positively regulates SA signaling by closing stoma in *Arabidopsis*. Therefore, we hypothesize that the function of alfalfa MPK9 is similar in *Arabidopsis* to defense freezing stress.

### 4.4 PAL pathway could be the dominant method of synthesizing SA in alfalfa against freezing stress

Plants use two independent pathways (ICS and PAL pathways) to synthesize SA. In *Arabidopsis*, Xu et al. (2017) demonstrated that the ICS pathway is the most common way to synthesize SA against biotic and abiotic stress, especially in pathogen-induced SA synthesis (Ding and Ding, 2020). Furthermore, ICS1 has been reported to play a more critical role than ICS2 in SA synthesis (Huang et al., 2020). In soybean, the PAL and ICS pathways contributed equally to SA biosynthesis (Shine et al., 2016). Ogawa et al. (2006) revealed that the PAL pathway was the primary route to the synthesis of SA in tobacco by investigating ICS and PAL gene expression. Therefore, the dominant pathway involved in SA synthesis depends on the plant species. In our study, we investigated the expression of ICS and PAL genes based on the RNA-seq database and WGCNA results. Unfortunately, there were no DEGs as ICS genes (**Figure 5**), which could be because the freezing stress time was too short. However, 11 PALs were identified as DEGs. Automatically, the PAL pathway could be dominant in alfalfa leaves under freezing stress, leading to the biosynthesis of SA and SA accumulation.

### 4.5 NPR1-dependent and independent pathway-related genes possibly participate in SA to resist freezing stress in alfalfa

Exogenous application of SA improving cold stress tolerance has been reported in different species, including maize, potato, *Aradibopsis* (Saleem et al., 2021b), rice (Wang et al., 2009b), wheat (Wang et al., 2021), cucumber, pepper, banana (Hara et al., 2011), and barley (Mutlu et al., 2016). However, numerous studies focused on the physiological changes and the expression of cold signaling genes (Miura and Tada, 2014), though how the SA signaling pathway regulates related genes to improve cold stress is unknown. SA signaling could regulate NPR1-dependent and NPR1-independent pathway-related genes to participate in resistance to biotic and abiotic stress (Li et al., 1999; Yu et al., 2001; An and Mou, 2011), and the NPR1-dependent pathway regulated more than 98% of SA-responsive genes (Saleem et al., 2021a). With NPR1 as the SA receptor, SA and the transcriptome coactivator can regulate SA-dependent gene expression against biotic and abiotic stress (Wu et al., 2012). In an NPR1-dependent pathway, SA could modulate signaling transducers, including MAPK signal cascades, to contribute to WRKY transcript factors binding to the W-box to promote the expression of NPR1 genes (Chai et al., 2014). As the concentration of SA increased, redox changes occurred and thioredoxin (TRx h) monomerized NPR1 to reduce NPR1 (Saleem et al., 2021a). When NPR1 enters the nucleus, it could bind TGA or WRKY transcript factors with the as-1 element or W-box to regulate SA-related gene expression (Wang et al., 2006; Saleem et al., 2021a). We identified 134 WRKY genes, 4 NPR1 genes, 21 TGA genes, 11 TRx h genes, and 4 PR1 genes in our study (**Figure S4**). Furthermore, to analyze which gene is dominant in this pathway, we used WGCNA to identify possible WRKY 22 and TGA1 that play vital roles in the SA signaling pathway. Numerous studies demonstrated that WRKY22 could be induced by abiotic and biotic stresses, including cold and pathogen stress (Zhang et al., 2015; Kloth et al., 2016; Li et al., 2018a; Li et al., 2019; Chung et al., 2020). Therefore, signal transduction pathways could convert common regulators-WRKY22. Previous studies have found that VvWRKY22 participates in sugar accumulation in grapes (Huang et al., 2021). WRKY22 of LA Hybrid Lily not only regulates gibberellin signaling but is also involved in low-temperature signals (Li et al., 2019), and Ms WRKY22 has been identified in pathogen attacks (Zhang et al., 2022). In *Arabidopsis*, *AtWRKY22* was involved in Pathogen-associated molecular patterns (PAMP)-triggered MAPK cascades (AtMEKK1-AtMKK4/5-AtMPK3/6), which induced the SA signaling pathway to regulate PR1 expression and resulted in expression of genes related to plant growth and cell-wall loosening (Zipfel et al., 2004; Kloth et al., 2016). Furthermore, Kloth et al. (2016) reported that abiotic stress induced WRKY22 by MAPK cascades or indirectly by SA accumulation. Therefore, WRKY22 is involved in the SA signaling pathway and the function of WRKY22 in alfalfa was similar to that in *Arabidopsis*.

TGA transcription factors bind to NPR1 to regulate the expression of PR genes (Kesarwani et al., 2007). In *Arabidopsis*, 10 TGAs were identified (Li et al., 2018b), while only TGA1-TGA7 could interact with NPR1. Furthermore, TGA2, TGA3, TGA5, TGA6, and TGA7 have been identified to interact with NPR1 in yeast and planta, but only TGA1 and TGA4 bind to NPR1 in SA-induced planta leaves (Despres, 2003; Fobert and Després, 2005; Kesarwani et al., 2007). Therefore, TGA1 was induced in our study (**Table 1**). Budimir et al. found that TGA1 was expressed in SA-induced NPR1-dependent genes (Budimir et al., 2020). Moreover, TGA1 interacts with NPR1 in SA, which regulates the expression of PR genes (Budimir et al., 2020). Sun et al. found that TGA1 regulates SA biosynthesis (Sun et al., 2017), and Kesarwani et al. (2007) found that TGA1 plays a dominant in basal resistance, which coincides with our results.

Except for the NPR1-dependent pathway, SA could induce the expression of NPR1-independent pathway-related genes against stress (Uquillas et al., 2004), including *GPXs*, *GSTs*, *HSPs*, *PODs*, *SODs*, *APXs*, GSHs, and *LEAs* (Aftab and Yusuf, 2021; Saleem et al., 2021b). *SODs*, *PODs*, *APXs*, *GSTs*, and *HSPs* were induced in our study (**Figure S4**). In numerous studies, SA could induce *SOD*, *POD*, APX, and *GST* encoding genes to upregulate the activities of related enzymes and alleviate freezing stress damage (Wang et al., 2020b; Serna-Escolano et al., 2021). In addition, the *WRKY* gene plays an important role in SA’s improved encoding genes of antioxidant enzymes in freezing stress (Yokotani et al., 2013; Wang et al., 2020b). Wan et al. (2009) found that low-temperature stress HSP73 could increase the accumulation of SA, while HSP70 and HSP90 have been demonstrated to accumulate under cold temperature stress and SA application. *HSPs* could prevent the proteins from denaturing and misfolding (Lopez-Matas et al., 2004). Therefore, SA application on alfalfa under freezing stress induces the expression of *SODs*, *PODs*, *APXs*, *GSTs*, and *HSPs* genes.

## 5 Conclusion

**Figure 6:**
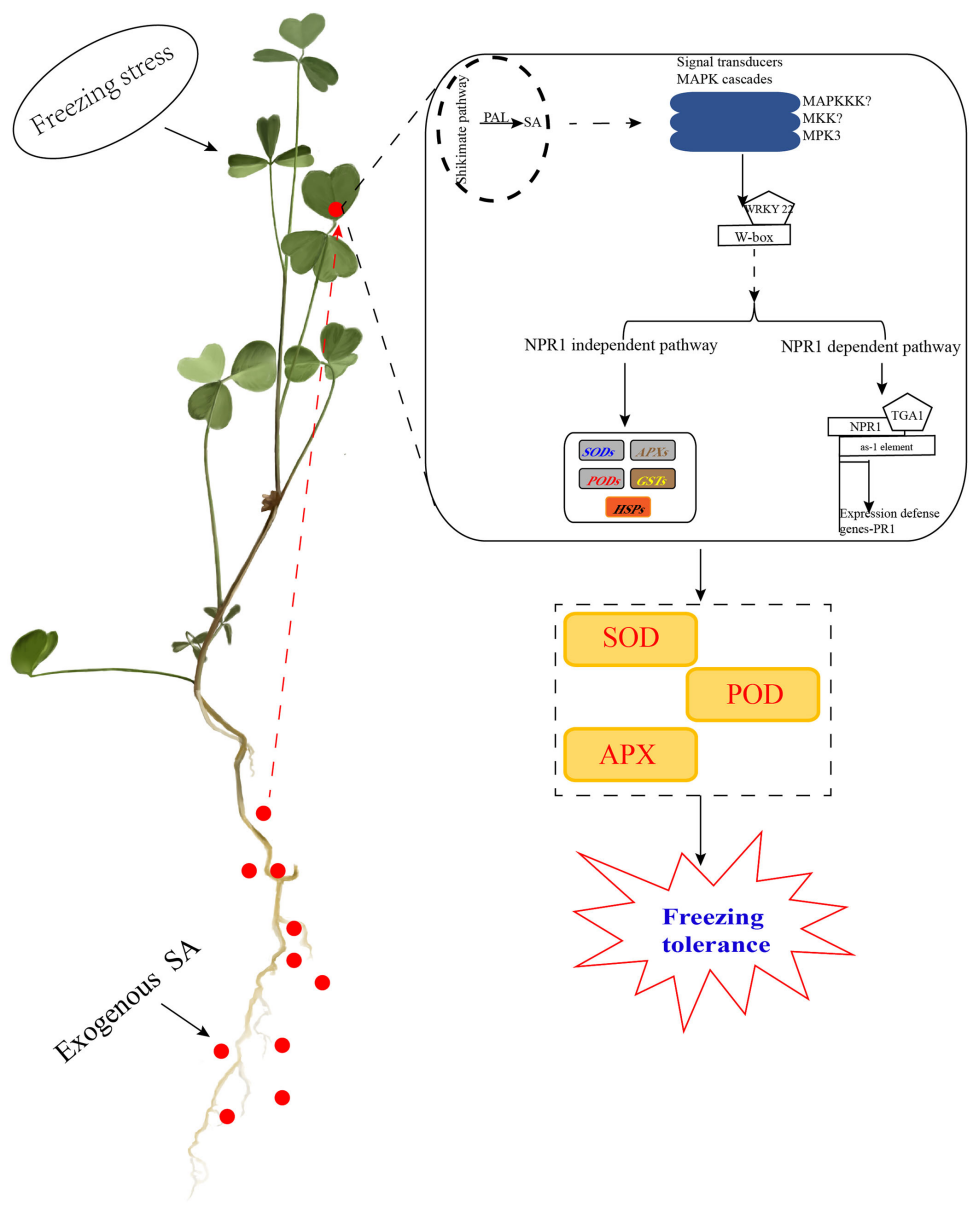
Working diagram for the mechanism of SA application improved alfalfa freezing tolerance in SA signaling transduction. Exogenous SA could improve the free SA in alfalfa leaves after freezing stress primarily through the PAL pathway. Moreover, SA induced MAPK cascades to regulate WRKY22 to participate in the SA signaling pathway, including the NPR1-dependent pathway and NPR1-independent pathway. We hypothesize that the hub gene MPK3 contributes to WRKY22 binding to the W-box to promote the expression of NPR1 genes downstream and NPR1-independent related genes against freezing stress. In the NPR1-dependent pathway, NPR1 enters the nucleus, which could bind to TGA1 with the as-1 element to regulate the expression of *PR1* genes to defend against freezing stress. In the NPR1-independent pathway, WRKY22 could directly regulate the expression of *SODs*, *PODs*, *APXs*, *GSTs*, and *HSPs*. Collectively, SA enhanced the antioxidant enzymes of SOD, POD, and APX to improve freezing tolerance in alfalfa.

In conclusion, exogenous SA can improve the accumulate of free SA in alfalfa leaves primarily through the PAL pathway under freezing stress. Moreover, SA induced MAPK cascades to regulate WRKY22 to participate in the SA signaling pathway, including the NPR1-dependent pathway and the NPR1-independent pathway, to regulated related genes against freezing stress. Interestingly, the transcriptome analysis and WGCNA database found that MPK3 could contribute to WRKY22 binding to the W-box and promote the downstream expression of NPR1 genes and NPR1-independent related genes to defend against freezing stress. In the NPR1-dependent pathway, NPR1 enters the nucleus, which could bind to TGA1 with the as-1 element to regulate the expression of PR1 genes to defend against freezing stress. In the NPR1-independent pathway, WRKY22 could directly regulate the expression of *SODs*, *PODs*, *APXs*, *GSTs*, and *HSPs*. Collectively, SA enhanced the antioxidant enzymes of SOD, POD, and APX to improve the survival of alfalfa under freezing stress. Furthermore, MPK9 can positively regulate SA signaling transduction to defend against freezing stress (**Figure 6**). Our study provides new insights into the mechanism of how exogenous SA can improve defense against freezing stress in alfalfa.

## Data availability statement

The data presented in the study are deposited in the NCBI repository, the accession number is PRJNA867517. 

## Author contributions

SS and XW conceived and designed research; XW, JM and WK conducted experiment; XW and JM analyzed data; XW, and WK wrote manuscript; XW, JM and SS finalized the manuscript. All authors contributed to the article and approved the submitted version.

## Supplementary material

Supplementary Figure 1 Numbers of differential upregulated and downregulated expressed genes in alfalfa samples. WCK-0, WCK-1, WCK-2, WCK-3, and WCK-4 represent the samples of 0 μM SA pretreatment seedlings exposed to -10°C at 0, 0.5, 1, 2 h, and allowed to recover for 2 days, respectively. T-0, T-1, T-2, T-3, and T-4 represent the samples of 200 μM SA pretreatment seedlings expose to -10°C at 0, 0.5, 1, 2 h, and allowed to recover for 2 days, respectively.

Supplementary Figure 2 The results of the DEGs in most upregulated (WCK-0-vs-WCK-4, T-0-vs-T-4) and downregulated (WCK-0-vs-WCK-3, T-0-vs-T-3) DEG comparisons mapped to the GO and KEGG databases. A1 and A2 represent the results of all the DEGs in the WCK-0-vs-WCK-4 comparison mapped to the GO and KEGG databases, respectively. B1and B2 represent the results of all the DEGs in the T-0-vs-T-4 comparison mapped to the GO and KEGG databases, respectively. C1 and C2 represent the results of all the DEGs in the WCK-0-vs-WCK-3 comparison mapped to the GO and KEGG databases, respectively. D1and D2 represent the results of all the DEGs in the T-0-vs-T-3 comparison mapped to the GO and KEGG databases, respectively.

Supplementary Figure 3 The GO and KEGG enrichment of the darkgreen **(A1, A2)**, darkgrey **(B1, B2)**, salmon **(C1, C2)**, and magenta **(D1, D2)** modules.

Supplementary Figure 4 The heatmap of expressed genes in the SA signaling pathway. **(A)** the expression genes of *MAPKKKs*; **(B)** the expression genes of *MKKs*; **(C)** the expression genes of *MPKs*; **(D)** the expression genes of *NPR1s*; **(E)** the expression genes of *TGAs*; **(F)** the expression genes of *TRx hs*; **(G)** the expression genes of *PR1s*; **(H)** the expression genes of *WRKYs*; **(I)** the expression genes of *SODs*; **(J)** the expression genes of *PODs*; **(K)** the expression genes of *APXs*; **(L)** the expression genes of *GSTs*; **(M)** the expression genes of *HSPs*.

Supplementary Figure 5 Expression profile of 6 candidate genes by qRT-PCR and RNA-seq. Error bar represents STEDV of each treatment.
