# Supplementary figures and images for: Exogenous application of salicylic acid improves freezing stress tolerance in alfalfa
Source: Front Plant Sci. 2023 Mar 9;14:1091077. doi: 10.3389/fpls.2023.1091077 (PMC10034032; doi:10.3389/fpls.2023.1091077)

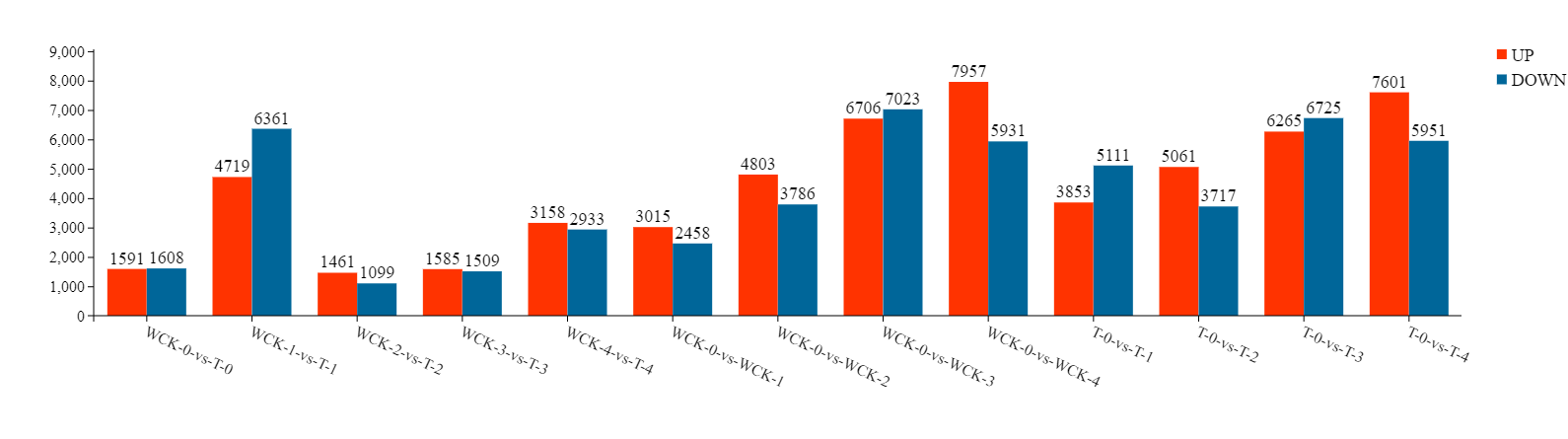

Supplement: Supplementary Figure 1 — Numbers of differential upregulated and downregulated expressed genes in alfalfa samples. WCK-0, WCK-1, WCK-2, WCK-3, and WCK-4 represent the samples of 0 μM SA pretreatment seedlings exposed to -10°C at 0, 0.5, 1, 2 h, and allowed to recover for 2 days, respectively. T-0, T-1, T-2, T-3, and T-4 represent the samples of 200 μM SA pretreatment seedlings expose to -10°C at 0, 0.5, 1, 2 h, and allowed to recover for 2 days, respectively. [file Image_1.png]

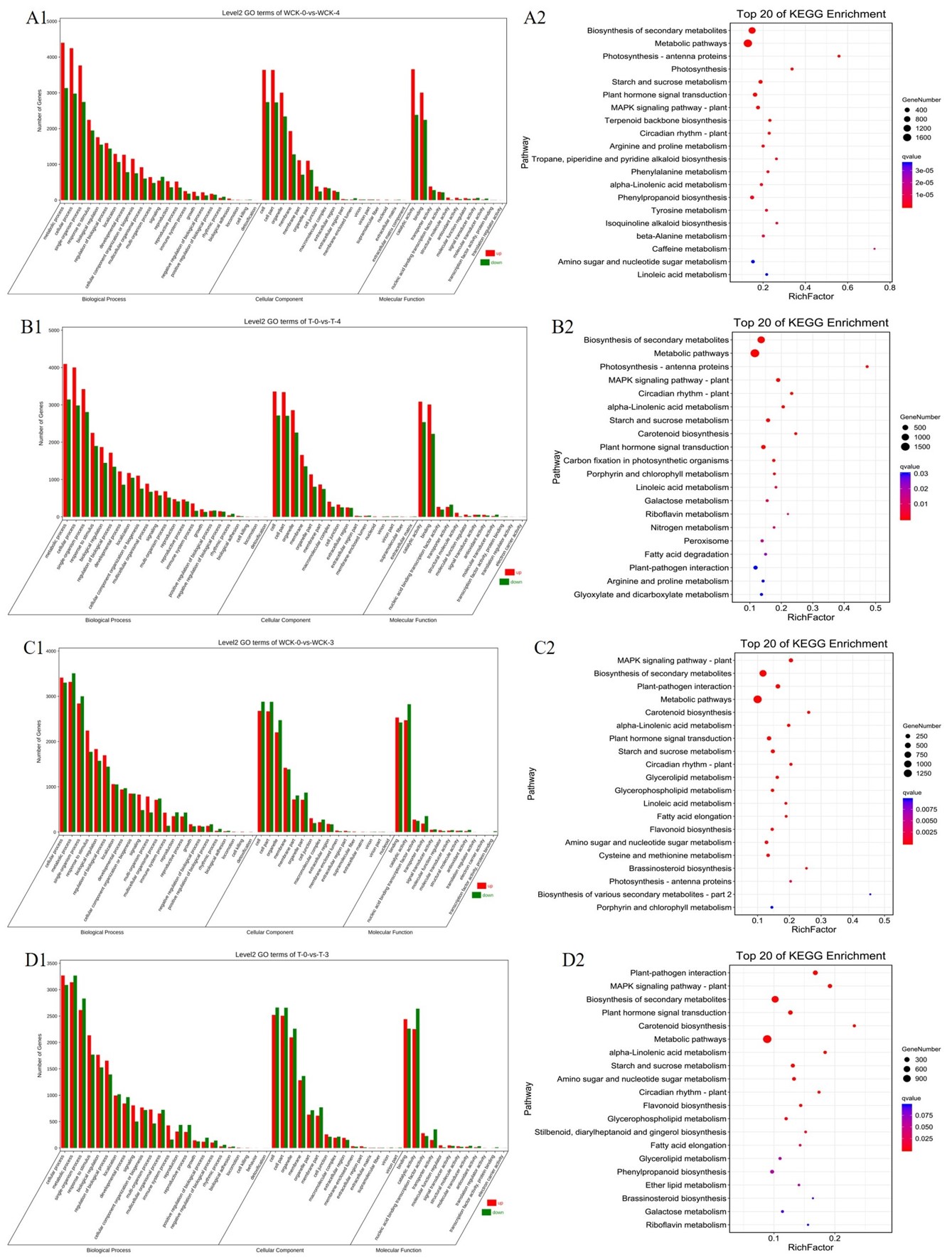

Supplement: Supplementary Figure 2 — The results of the DEGs in most upregulated (WCK-0-vs-WCK-4, T-0-vs-T-4) and downregulated (WCK-0-vs-WCK-3, T-0-vs-T-3) DEG comparisons mapped to the GO and KEGG databases. A1 and A2 represent the results of all the DEGs in the WCK-0-vs-WCK-4 comparison mapped to the GO and KEGG databases, respectively. B1and B2 represent the results of all the DEGs in the T-0-vs-T-4 comparison mapped to the GO and KEGG databases, respectively. C1 and C2 represent the results of all the DEGs in the WCK-0-vs-WCK-3 comparison mapped to the GO and KEGG databases, respectively. D1and D2 represent the results of all the DEGs in the T-0-vs-T-3 comparison mapped to the GO and KEGG databases, respectively. [file Image_2.jpg]

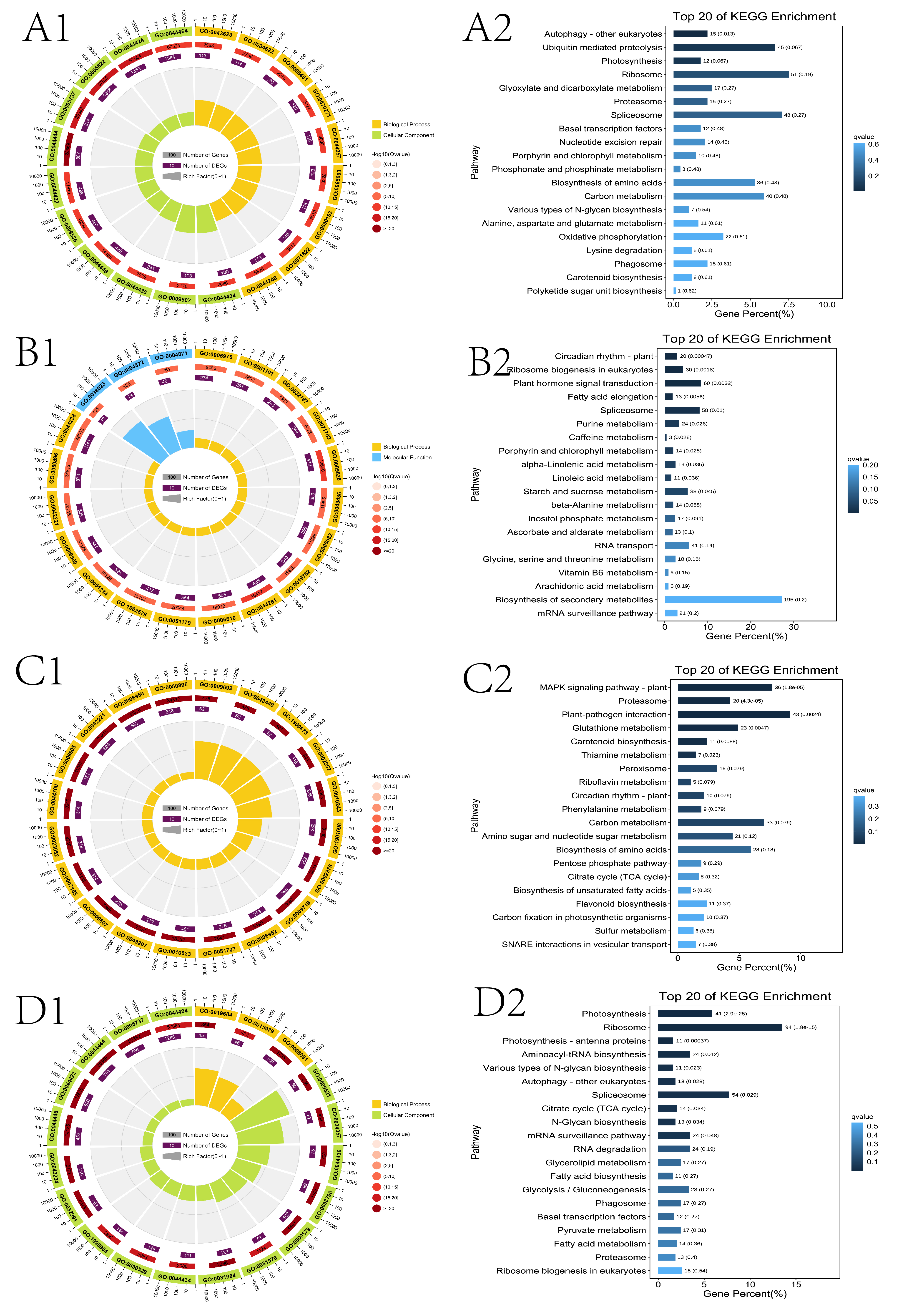

Supplement: Supplementary Figure 3 — The GO and KEGG enrichment of the darkgreen (A1, A2), darkgrey (B1, B2), salmon (C1, C2), and magenta (D1, D2) modules. [file Image_3.jpg]

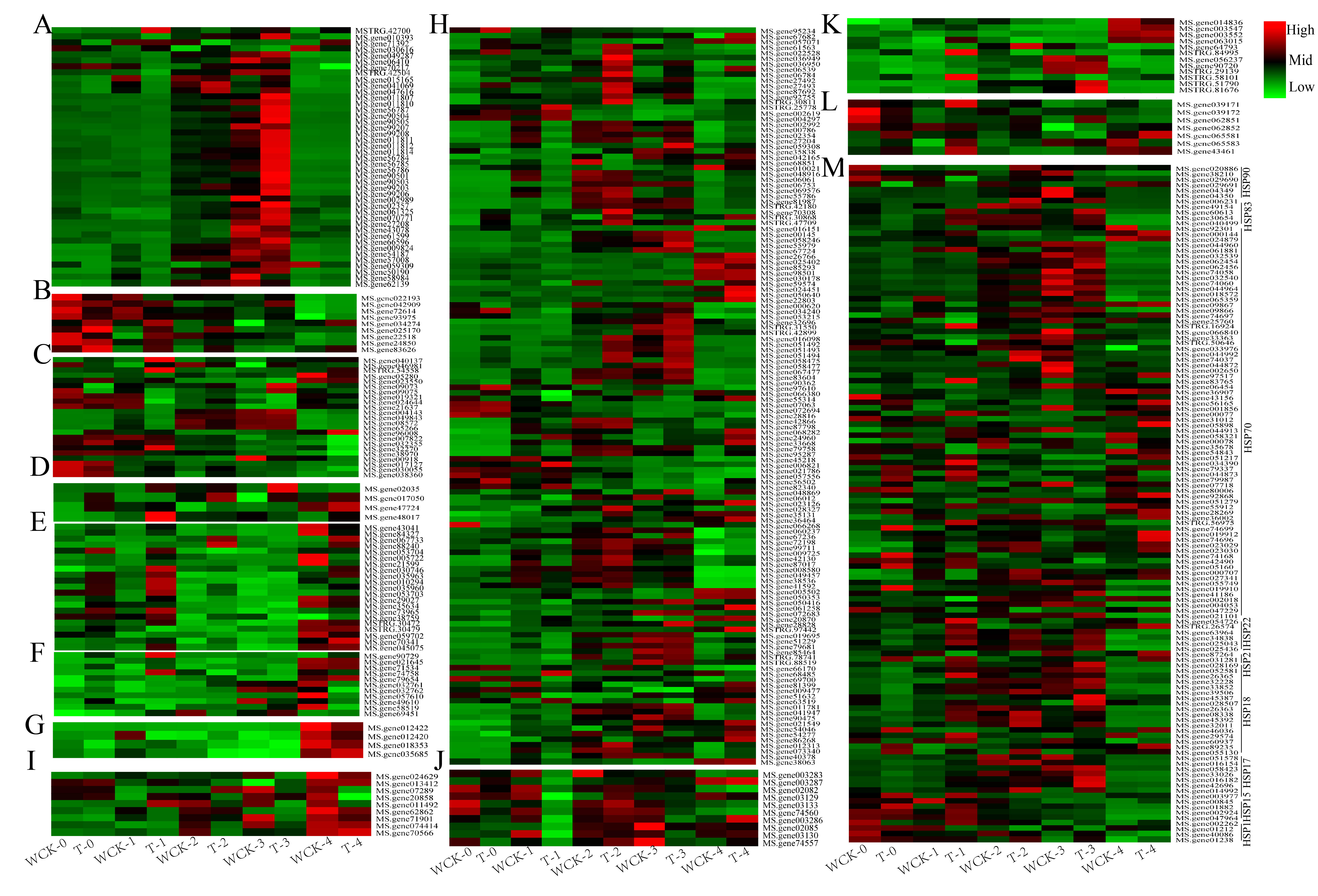

Supplement: Supplementary Figure 4 — The heatmap of expressed genes in the SA signaling pathway. (A) the expression genes of MAPKKKs; (B) the expression genes of MKKs; (C) the expression genes of MPKs; (D) the expression genes of NPR1s; (E) the expression genes of TGAs; (F) the expression genes of TRx hs; (G) the expression genes of PR1s; (H) the expression genes of WRKYs; (I) the expression genes of SODs; (J) the expression genes of PODs; (K) the expression genes of APXs; (L) the expression genes of GSTs; (M) the expression genes of HSPs. [file Image_4.jpg]

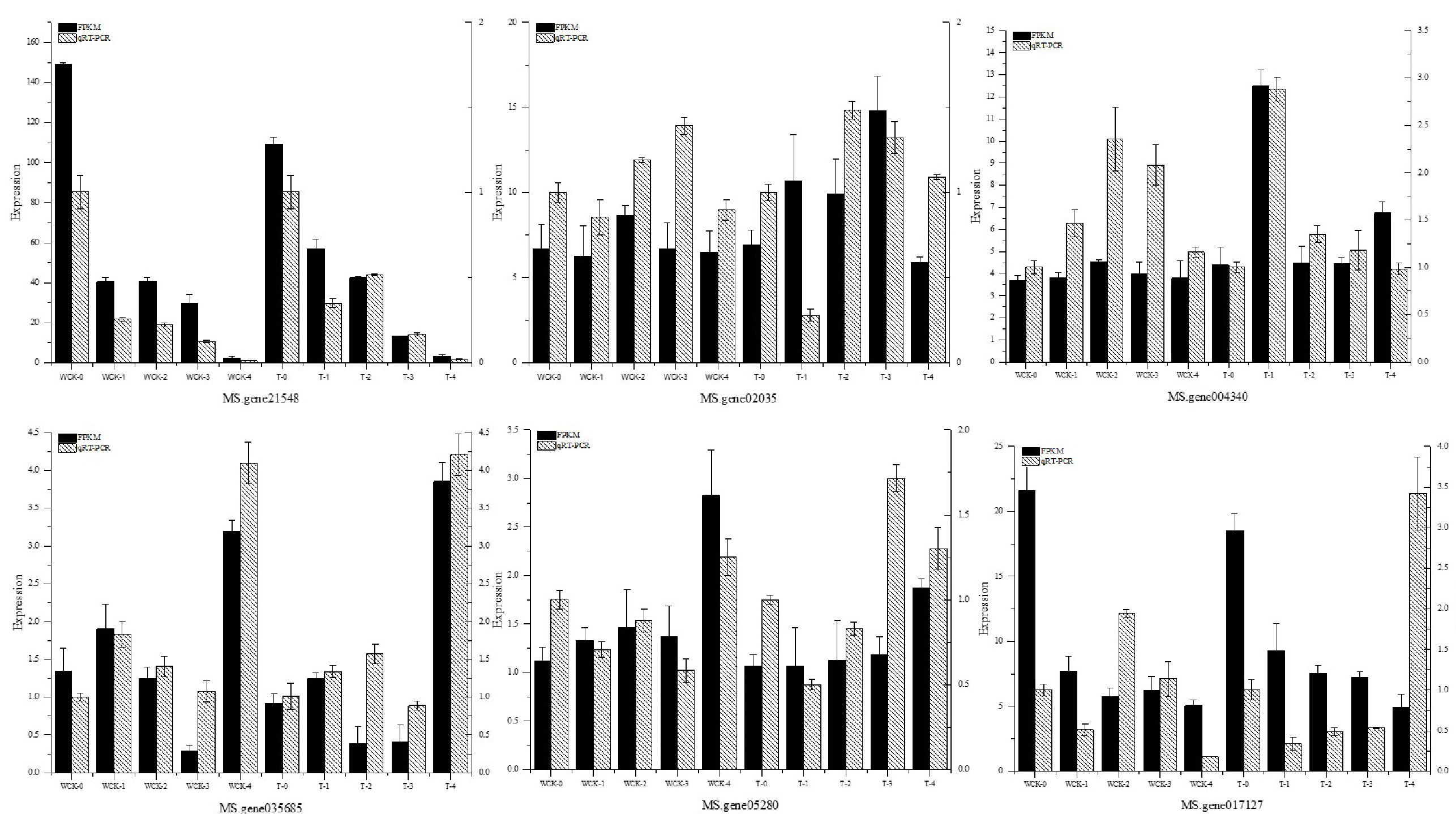

Supplement: Supplementary Figure 5 — Expression profile of 6 candidate genes by qRT-PCR and RNA-seq. Error bar represents STEDV of each treatment. [file Image_5.jpg]
